# Lung cancer screening completion rates and neighborhood disadvantage among primary care patients in an integrated delivery network health system: a retrospective data analysis

**DOI:** 10.1186/s12875-026-03395-2

**Published:** 2026-05-27

**Authors:** Anu Sangraula, Shannon Melvin, Traci N. Bethea, Mindi Messmer, George Luta, Randi M. Williams

**Affiliations:** 1 Cancer Prevention and Control Program, Lombardi Comprehensive Cancer Center, Georgetown University Medical Center, Washington, DC USA; 2 Department of Oncology, Georgetown University Medical Center, Georgetown University, Washington, DC USA; 3 Center for Biostatistics, Informatics, and Data Science, MedStar Health Research Institute, Washington, DC USA; 4 Department of Biostatistics, Bioinformatics, and Biomathematics, Georgetown University Medical Center, Washington, DC USA

**Keywords:** Lung cancer screening, Primary care, Area deprivation index, Neighborhood disadvantage

## Abstract

**Background:**

In the United States, lung cancer screening (LCS) via low-dose computed tomography is recommended for adults at high risk, but LCS uptake is poor, particularly among patients with lower socioeconomic status (SES). We evaluated neighborhood disadvantage, a measure of SES, as a predictor of LCS completion rates among primary care patients seen in the Washington, D.C. and Maryland area to understand how neighborhood disadvantage may impact LCS completion.

**Methods:**

Utilizing electronic health record data, we calculated LCS completion rates in the past 12 months among patients who received an LCS order (*N* = 829) to a hybrid LCS program between September 2022 and August 2023 at 51 primary care clinics. To measure neighborhood disadvantage, we used the census block level Area Deprivation Index (ADI), a composite index of demographic and socioeconomic variables. The state-specific ADI decile is ranked on a scale of 1 to 10, with a higher score reflecting greater neighborhood disadvantage. A logistic regression model with generalized estimating equations (GEE) assessed the association of ADI with LCS completion, controlling for race, smoking status, minutes required to travel to the nearest LCS screening location, and clinic type.

**Results:**

Most potentially eligible patients who received a LCS order (766, 92.4%) had a standardized address that could be linked to ADI data. The mean age was 66 (standard deviation = 7) years, 35% were Black or African-American, and 65% were currently smoking. Among patients ordered an LCS, the overall completion rate was 24%. The ADI was inversely associated with LCS completion: a one decile increase in ADI corresponded to significantly lower odds of completing LCS (odds ratio = 0.91, 95% confidence interval: 0.86–0.97).

**Conclusions:**

In a large, socioeconomically diverse health system, higher neighborhood disadvantage was associated with lower completion among patients ordered an LCS. While a LCS order is necessary to access LCS, strategies that consider neighborhood context are needed to increase successful completion of LCS among lower SES groups. Primary care practices may be an effective setting for these strategies due to their focus on cancer screenings and role as often being the first point of contact for LCS.

This study was approved by the Georgetown-MedStar Institutional Review Board (STUDY00009269).

**Supplementary Information:**

The online version contains supplementary material available at 10.1186/s12875-026-03395-2.

## Background

Lung cancer is the leading cause of cancer-related mortality in the United States (U.S.), with more than 125,000 deaths expected in 2026 [1]. Previous lung cancer screening (LCS) trials, including the National Lung Screening Trial and Nederlands–Leuvens Longkanker Screenings Onderzoek (NELSON) trial, showed that LCS via low-dose computed tomography reduced lung cancer mortality by 20–24% [2, 3]. Due to this, the U.S. Preventive Services Task Force currently recommends annual LCS for asymptomatic high-risk individuals, however, utilization is poor [4]. The causes of low utilization of LCS are multifactorial including individual-level factors like low socioeconomic status (SES) [5–9].

Primary care serves an essential role in the uptake of LCS because primary care providers are often the first point of contact in the health care system for many patients [10, 11] and are responsible for identifying screening-eligible patients, providing information regarding screening, facilitating shared decision making, placing the order, and following up on results [12, 13]. While an LCS order is necessary, it may not be sufficient to increase LCS rates. There is evidence that even after receiving an order some populations, including lower income individuals, may encounter more barriers to completing LCS, which results in missed opportunities to diagnose lung cancer at earlier stages [14–16].

Previous studies on LCS uptake have focused on the role of health system-, provider-, and patient-level characteristics [16–19], however, additional efforts are needed to understand how neighborhood context may influence individual LCS behavior and to support primary care practices in addressing barriers this context may pose. For instance, prior literature for other cancer screenings highlight how neighborhood disadvantage may be associated with screening behavior [20–23]. Although area-level differences, such as rurality, have been examined to evaluate access to LCS [24–27], few studies have focused on how neighborhood disadvantage may impact LCS [9, 28]. To improve LCS utilization, an increased understanding of neighborhood context is needed in order to develop targeted interventions that will support LCS completion and guide primary care clinics in implementing these strategies.

The Area Deprivation Index (ADI) is a scientifically validated measure that ranks neighborhoods by disadvantage at the state and national level [29]. Numerous studies using the ADI have demonstrated an association with cancer screening outcomes [30–33]. To our knowledge, the present study is the first to assess the association of the ADI with LCS completion among primary care patients. Thus, our study aimed to examine neighborhood disadvantage as a predictor of LCS completion rates among primary care patients utilizing electronic health record (EHR) data from an integrated delivery network health system and the largest healthcare provider in Maryland and the District of Columbia (D.C.) metropolitan region [34].

## Methods

### Setting

Currently, this integrated delivery network health system consists of 10 hospitals, 60 primary care clinics, and multiple other sites of care across D.C., Maryland, and Virginia. Primary care includes over 320 providers who are physicians, physician assistants, and nurse practitioners. This study included 51 primary care clinics in D.C. and Maryland. At the time of data collection, this integrated delivery network health system had 56 primary care clinics. Five pediatrics clinics were excluded from the analysis. This mid-Atlantic health system uses the Oracle Cerner EHR and it delivers LCS through a hybrid approach, with two hospital-based centralized programs and decentralized sites throughout the rest of the system.

### Study population

We conducted a retrospective data analysis utilizing EHR data from September 1, 2022 to August 31, 2024. The inclusion criteria were: individuals who had a primary care clinic visit and who were between the ages of 50–80 years at the time of the appointment, had a smoking history (i.e., currently smoking or formerly smoked), and did not have a previous lung cancer diagnosis. Due to the high missingness of pack years (85% missing), years since quit, duration, and other variables used to determine LCS eligibility, further exclusion based on available smoking history data was not feasible and this this resulted in a sample of patients who were potentially eligible for LCS. We excluded patients residing in Virginia and other states as the health system is primarily concentrated in the D.C. and Maryland regions. This study was approved by the Georgetown-MedStar Institutional Review Board (STUDY00009269) and the need to consent was waived.

### Data elements

#### Demographic and clinical characteristics

Race, sex, age, residential address, and insurance type were extracted from the EHR and used to characterize the sample. The primary care clinic location was included in our dataset, allowing us to determine the type of primary care clinic (community-based vs. hospital-based).

#### Smoking history

Where available, we included packs per day, duration (age started smoking; age stopped smoking (if formerly smoked), and pack years (total packs per day smoked multiplied by total years smoked) from the social history form in the EHR.

#### Lung cancer screening eligibility

Due to the high missingness of smoking-related data within the EHR [35, 36], we were unable to determine how many patients were eligible for lung cancer screening according to the U.S. Preventive Services Task Force eligibility criteria (i.e., 50–80 years old, 20 pack years or more, currently smoking or have quit within 15 years). Of the patients with available data, we categorized packs per day, duration, and pack years to determine the distribution. There was no missingness for smoking status as having a previous smoking history was a requirement to be included in the dataset. For patients who were ordered a LCS, we abstracted smoking status (yes, currently smoking vs. no, not currently smoking) and if the patient discussed smoking cessation with the provider (yes vs. no) from the LCS order form. In this integrated delivery network health system, an LCS recommendation fires annually on January 1st for individuals who are 50–80 years of age with an ever-smoking history to prompt the provider to assess smoking history and LCS eligibility.

#### Area deprivation index

The ADI is a scientifically validated measure of neighborhood disadvantage that was last updated in 2023 and calculates a score based on U.S. Census American Community Survey 5-Year Data related to income, housing, education, and occupation and ranks neighborhoods using state-based and national percentiles [29]. The ADI has been used in prior studies that have abstracted data from the EHR [37]. We used the state-specific ADI deciles as it ranks a neighborhood’s socioeconomic disadvantage against other census block groups within a specific state. The ADI was included as a continuous variable in the Generalized Estimating Equations (GEEs). Residential addresses of the patients were abstracted from the EHR. We used the Census Geocoder and the “Public_AR_Current” benchmark to match patients’ addresses with geographic identifiers.

#### LCS order and completion (Primary Outcome)

LCS order was defined as having the scan ordered (yes vs. no) in the first 12 months of the data period (September 1, 2022 to August 31, 2023). LCS completion rate was defined as having the scan completed (yes vs. no) within the following 12 months among patients who received a LCS order. The 12-month timeframe was chosen because LCS orders do not expire until 12 months after the order is placed in the health system. We generated a screening rate for each decile of ADI by dividing the number of orders placed in each decile by the number of completions. This was then converted to a percent.

#### Distance to screening location

We identified locations of LCS radiology sites in D.C. and Maryland using the LCS locator tool provided by the American College of Radiology (ACR) [38, 39]. We calculated Euclidean distance and road network distance using ArcGIS Pro (Version 3.4) [40]. We also calculated the number of minutes traveled when calculating road network distance as a complementary measure of distance.

### Statistical analysis

To assess the association between neighborhood deprivation and LCS completion with consideration of covariates, we first conducted bivariate analyses using chi-square and ANOVA tests. A t-test was also performed to compare those who complete the LCS with those who did not with regard to the means of continuous variables that had a significance association with completion. In multivariable analyses, we accounted for clustering at clinic level by using a GEE approach. We used a logistic regression model with GEE to examine the association of LCS completion with ADI as the primary predictor. Based on the results of the bivariate analyses, variables that were found to be significantly associated with LCS completion (*p* < 0.05) in the bivariate analysis were included in the logistic regression model with GEE. An exchangeable working correlation structure was employed and Wald chi-square tests were used to determine the significance of results. To evaluate the presence of a linear effect of ADI (on the log odds scale) we first treated it as a categorical variable (with 10 levels) and the log odds were plotted; the results supported linearity. As such, ADI was included as a continuous variable in the final model.

Results are presented as estimated odds ratios (ORs) with corresponding 95% confidence intervals (CIs). IBM SPSS (Version 29) [41] was used for the analysis; ArcGIS was used for generating maps to visually display data. A significance level of 0.05 was used to determine statistical significance.

## Results

### Population characteristics

From September 1, 2022 and August 31, 2023, *N* = 28,738 patients between the age of 50–80, with an ever-smoking history, and no previous lung cancer diagnosis were seen at least once for a primary care appointment in this integrated delivery health network. The mean age was 63 (standard deviation (SD) = 8) years, 32% were Black or African-American, and, using the most recent smoking status from the EHR, 10% were currently smoking. Of the 28,738, only 829 had an LCS order. We were not able to use data regarding total pack-years or years quit to confirm smoking history eligibility among patients with an LCS order. It is possible that some patients with an LCS order were ineligible or that confirmation of a patient’s LCS eligibility was documented elsewhere in the medical record such as in the clinical notes. Of those patients who had received an order, 766 had an address in the EHR that could be linked to the ADI measure and lived in D.C. or Maryland. The mean age was 65 (SD = 7) years and 35% were Black or African-American.

Of the 766 patients, 41% had smoking duration data available, 2.5% had a stop age, 71% had packs per day, and 20% had total pack years documented in the EHR based on their last recorded smoking history (Table 1). For the last recorded smoking status from the EHR, 54% were listed as currently smoking, 41% as formerly smoked, and 5% as having denied smoking. However, the 5% who denied smoking at their last visit previously had current or former listed as their smoking status due to our requirement of having an “ever smoked” history. All patients had a smoking status (65% currently smoking, 35% formerly smoked) listed in their LCS order form as it is a required field. Of those who were listed as currently smoking on their order form, *n* = 463 (93%) had discussed smoking cessation with their provider. Among patients ordered a LCS, the overall completion rate was 24%.

**Table 1:** Data availability for last recorded smoking history variables from EHR among 766 participants

Smoking-related Variables	LCS Completion Status				
	Incomplete (*n* = 585, 76%)		Complete (*n* = 181, 24%)		Total (*N* = 766)
	*N*	%	*N*	%	*N*
LCS Order Form Status - Currently Smoking?*					
Yes	400	80%	100	20%	500
No	185	70%	81	30%	266
Smoking status from EHR					
* Current*	333	80%	84	20%	417
* Denies*	30	86%	5	14%	35
* Former*	222	71%	92	29%	314
Availability of years smoked					
* Data missing*	360	80%	89	20%	449
* Data available*	225	71%	92	29%	317
Years smoked among documented					
* Less than 10 years*	7	88%	1	13%	8
* 10–19 years*	11	79%	3	21%	14
* 20–29 years*	25	56%	20	44%	45
* 30 or more years*	182	73%	68	27%	250
Availability of stop age					
* Data missing*	570	76%	177	24%	747
* Data available*	15	79%	4	21%	19
Stop age among documented					
* < 50 years old*	2	67%	1	33%	3
* ≥ 50 years old*	13	81%	3	19%	16
Availability of packs per day					
* Data missing*	169	76%	54	24%	223
* Data available*	416	77%	127	23%	543
Packs per day among documented					
* < 1 PPD*	167	76%	52	24%	219
* 1 < 2 PPD*	196	77%	58	23%	254
* ≥ 2 PPD*	53	76%	17	24%	70
Availability of pack years					
* Data missing*	481	79%	128	21%	609
* Data available*	104	66%	53	34%	157
Pack years among documented					
20–30 pack years	3	60%	2	40%	5
> 30 pack years	101	66%	51	34%	152

*p < 0.05

Black patients were more likely to complete LCS than White patients (30% vs. 19%, *p* = 0.002), as were patients who formerly smoked compared to patients who currently smoke (30% vs. 20%, *p* = 0.001) (Table 2). Patients seen at hospital-based clinics were more likely to complete their LCS than patients seen at community-based clinics (28% vs. 21%, *p* = 0.031). Furthermore, a higher ADI decile (*p* = 0.008), more minutes to travel to the nearest LCS site (*p*=0.024), and a greater Euclidean distance in miles (*p* < 0.001) were associated with not completing LCS. Supplemental Material 1 includes the distribution of patient characteristics and continuous area-level variables by category of ADI to provide additional context for the covariates.

**Table 2:** Distribution of participant characteristics, overall and by LCS completion status among 766 patients

Characteristics	Completion Status				
	Incomplete (*n* = 585, 76%)		Complete (*n* = 181, 24%)		
	*N*	%	*N*	%	Total (*N* = 766)
Age, M (SD)	66 (6)		65 (7)		65 (7)
Sex					
Female	293	76%	95	24%	388
Male	292	77%	86	23%	378
Race**					
Black	188	70%	80	30%	268
White	362	81%	85	19%	447
Other	35	69%	16	31%	51
Insurance					
Government/Medicaid/Medicare/MCO	223	76%	70	24%	293
Commercial/Private	336	78%	97	22%	433
Self pay/other	12	80%	3	20%	15
Missing	14		11		25
Clinic Type					
Hospital-based/Resident Clinic	176	72%	70	28%	246
Community-based	409	79%	111	21%	520
State of Residence					
Maryland (MD)	518	83%	108	17%	626
District of Columbia (DC)	67	48%	73	52%	140
Area-level Variables					
ADI Score (State-specific), M (SD) [1–10]***	6 (3)		5 (3)		6 (3)
Low (1–4)	186	71%	76	29%	262
Medium (5–7)	177	78%	51	22%	228
High (8–10)	222	80%	54	20%	276
Euclidean Distance (Miles), M (SD)**	6 (3)		5 (4)		6 (4)
Road Network Distance (Miles), M (SD)	7 (4)		7 (6)		7 (4)
Travel Minutes to Nearest LCS Site, M (SD)***	21 (9)		18 (9)		20 (9)

*p < 0.1, **p < 0.5, ***p < 0.001

### ADI and LCS completion

ADI was inversely associated with LCS completion: a one unit increase in ADI corresponded to significantly lower odds of completing LCS (OR = 0.91; 95% CI: 0.86–0.97; *p* = 0.004) (Table 3). Black patients (vs. white patients) had higher odds of completing their order (OR = 1.94; 95% CI: 1.33–2.83; *p* = 0.001). Patients who were not currently smoking (vs. currently smoking) had higher odds of completing their order (OR = 1.85; 95% CI: 1.30–2.64; *p* = 0.001). Finally, compared to patients with a shorter travel time in minutes to a LCS site, patients with a longer travel time to a LCS site had significantly lower odds of completing LCS (OR = 0.97; 95% CI: 0.95–0.99; *p* = 0.013).

**Table 3:** Results for the adjusted logistic regression model with GEE

	OR	95% CI	*p*-value
Area-level variables			
ADI State Decile	0.91	0.85–0.97	0.004
Travel Minutes to Nearest LCS Site	0.97	0.95–0.99	0.013
Smoking Status from LCS order (reference group: Yes, currently smoking)			
No (not currently smoking)	1.85	1.29–2.64	0.001
Race (reference group: White)			
Black	1.94	1.33–2.83	0.001
Other	1.54	0.78–3.02	0.210
Clinic location type (reference group: Community-based)			
Hospital-based	1.46	0.99–2.14	0.052

### LCS completion by state ADI

**Fig. 1:**
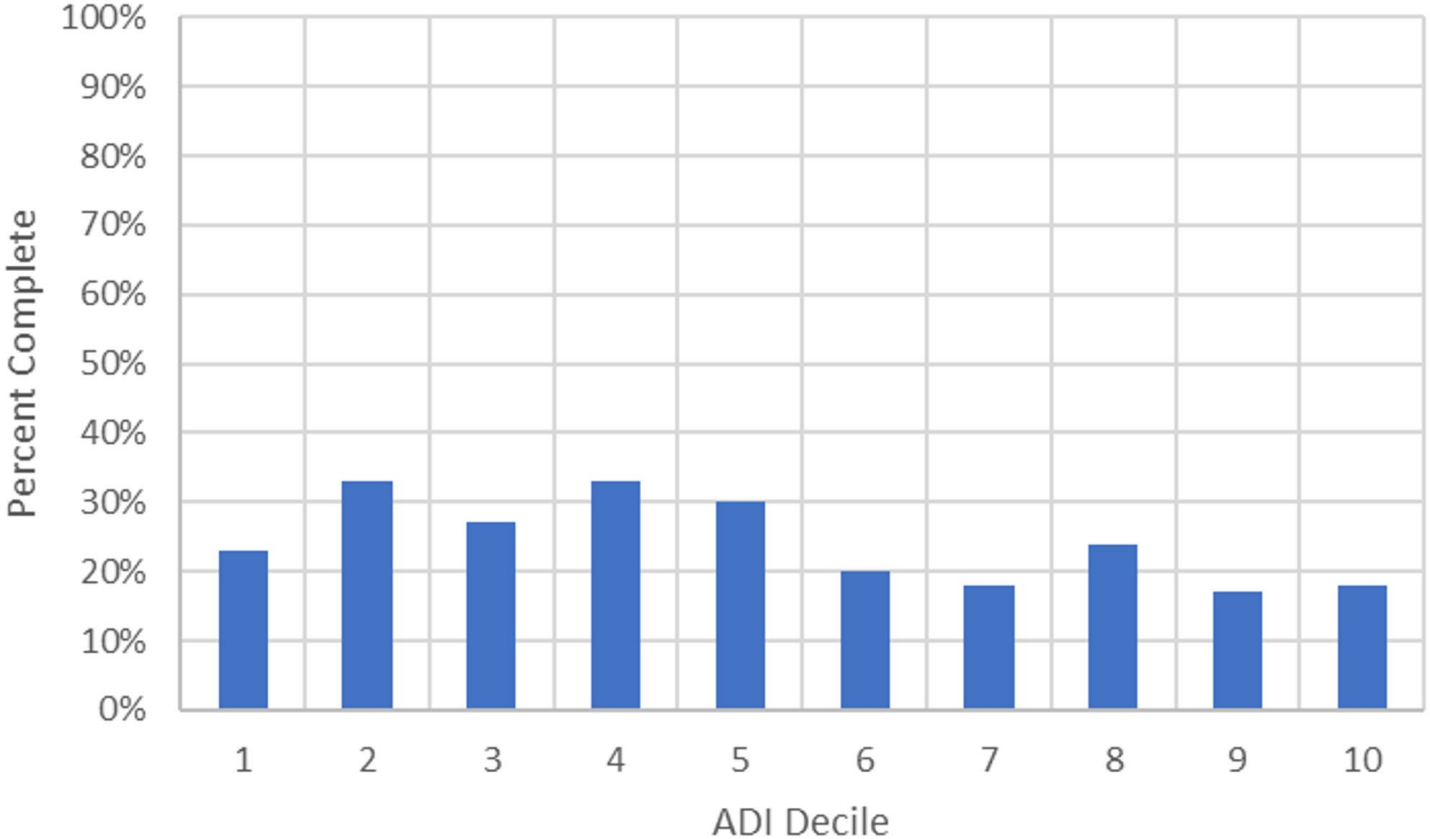
LCS completion percentages among patients with an order by ADI decile

A Pearson’s correlation revealed that LCS was inversely associated with ADI (*r*=-0.695, *p* = 0.026), as the percentage of scans completed was higher in Census blocks with lower neighborhood disadvantage compared to areas with higher neighborhood disadvantage (Fig. 1).

**Fig. 2:**
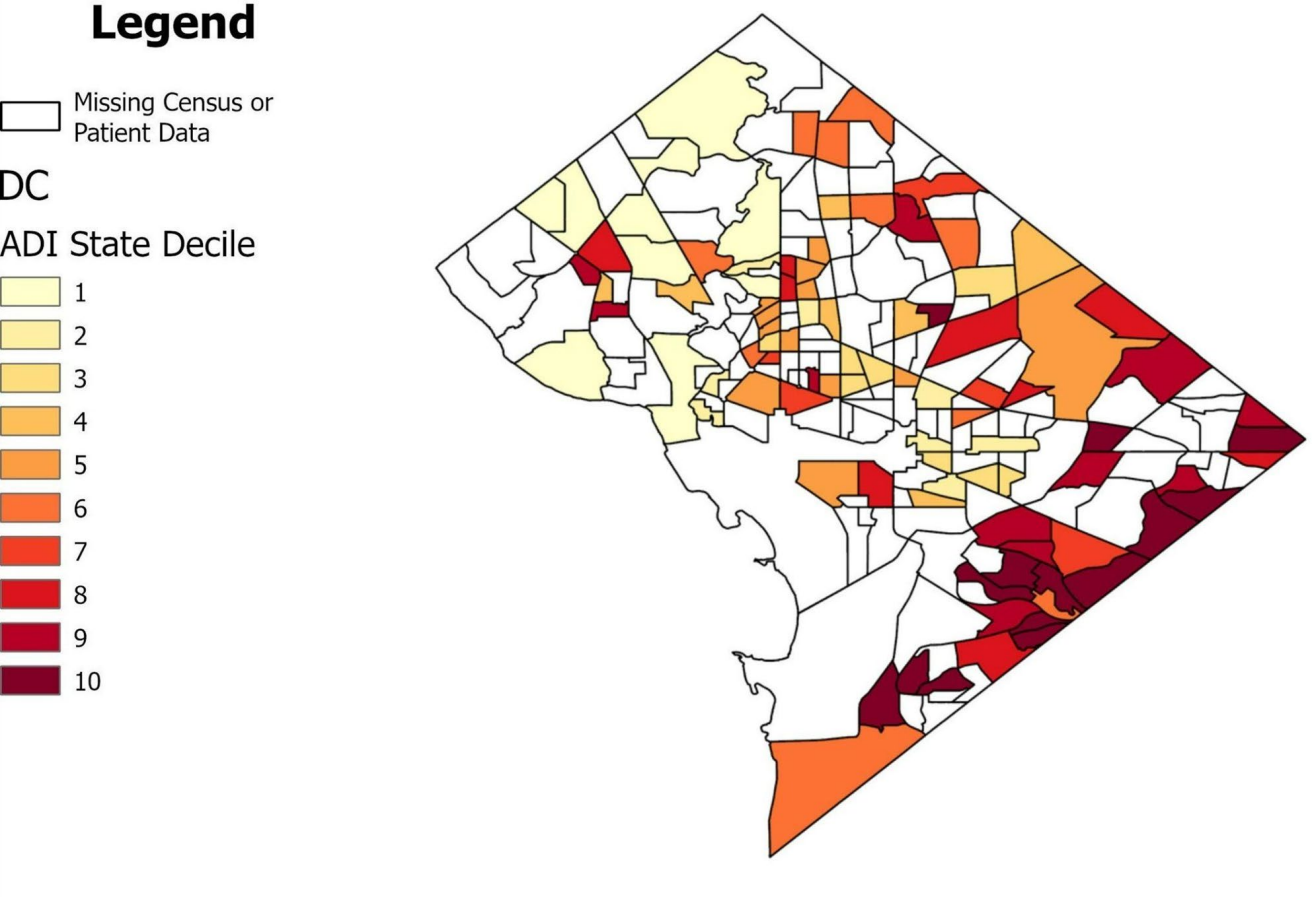
Distribution of state-specific ADI in DC by Census block group

As shown in Fig. 2, ADI was not evenly distributed across D.C., with higher ADI (i.e., greater neighborhood disadvantage) observed in neighborhoods located in the southern part of the city.

**Fig. 3:**
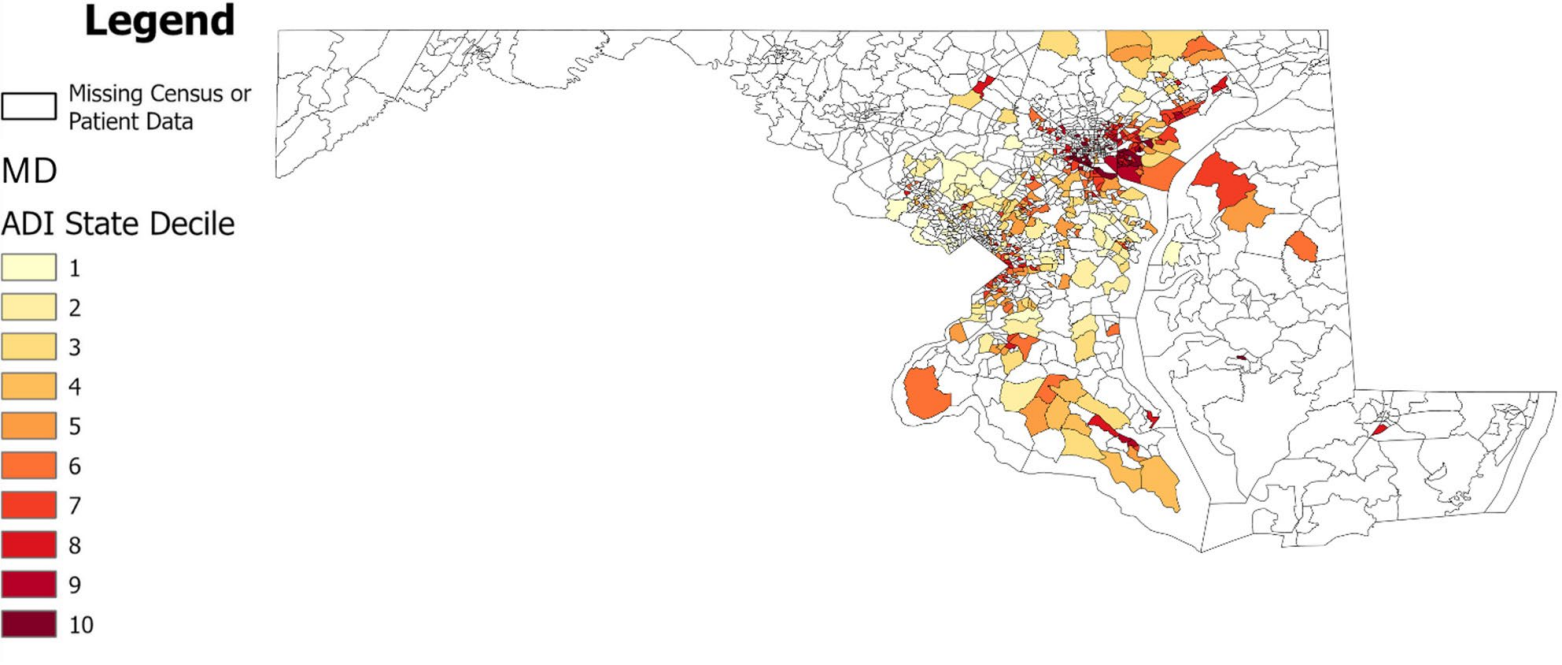
Distribution of state-specific ADI in MD by Census block group

As shown in Fig. 3, while 82% of the sample resided in Maryland, 90% of the block groups had zero patients or were not given an ADI score.

## Discussion

The aim of this retrospective data analysis was to examine if neighborhood disadvantage was associated with LCS completion among primary care patients with an order, in addition to other factors (Table 2). We found that, of those with an order, only 24% of patients completed LCS which is consistent with national LCS rates of approximately one in five eligible individuals undergoing LCS [42]. Our analysis found that the percentage of scans completed by Census block were correlated with neighborhood disadvantage (Fig. 1), and that patients living in higher deprivation areas had lower odds of completing the LCS order (Table 3). This finding is consistent with prior research [8, 9] and highlights how patients living in neighborhoods with high disadvantage may encounter more barriers to completing LCS. However, unlike our study, the patients referred to LCS in the Kim et al. and Neslund-Dudas et al. articles were evaluated before the United States Preventive Services Task Force updated their guidelines in 2021 [43]. Both studies also utilized the Yost index, a different validated neighborhood-level measure that encompasses seven neighborhood-level domains such as household income, occupation, and education [44, 45]. Additionally, Kim et al. specifically focused on adherence to annual LCS.

Our finding of patients who lived in higher deprivation areas having lower odds of completing their LCS order may also be related to our finding of patients who had a longer travel time to their nearest LCS site having significantly lower odds of completing their LCS (Table 3). Prior research supports how access to LCS sites via geographic proximity impacts lung cancer outcomes [26, 46, 47]. As such, referring patients to the nearest LCS site that accepts their insurance, rather than providing a general order to any LCS site, may help increase LCS completion. Potential interventions to increase LCS completion among patients who live in a highly disadvantaged area could include EHR modifications that would allow providers to easily find a LCS site closest to the address listed in the patient’s chart or order to a centralized LCS program that is led by trained staff who can support scheduling and offer closer tracking of LCS completions.

Patients who formerly smoked had higher odds of completing their LCS order in our study (Table 3), which underscores the importance of providing support to patients who are currently smoking to complete their LCS order. It may be especially impactful for primary care providers to address fear or reticence and stigma to complete the scan for patients who are continuing to smoke when making the order. While many interventions aim to help currently smoking LCS patients quit smoking [48], these interventions could also serve to help patients complete their LCS order. Understanding how to increase LCS uptake and completion is an active area of research. Recent meta-analyses [49–51], reviewed a variety of interventions aimed at increasing LCS orders and completion. The meta-analyses showed that patient navigation may be an effective strategy to improve LCS uptake among high-risk individuals [49] by addressing individual- and area-level barriers such as transportation [50, 52].

Additionally, in our sample, Black patients were more likely to complete their LCS order than White patients (Table 3). While this differs from findings in some studies [53, 54], it is consistent with others [55], which further underscores the importance of looking at variables beyond race to understand LCS utilization by subpopulations. This study suggests both individual-level factors and neighborhood context impact LCS uptake. Future quality improvement efforts in the primary care setting should consider the neighborhoods of their patients in order to increase uptake of LCS following an order, while also including a qualitative component to allow for a better understanding of how patients experience their neighborhood context and how it may hinder or facilitate completion of an LCS order. Due to the retrospective nature of this study, we were unable to include a qualitative component which could have provided additional context for our findings. However, previous qualitative studies describe how patients express facing barriers to LCS order completion including the fear of being diagnosed with cancer, cost of LCS, and scheduling their LCS [56, 57].

### Strengths and limitations

Our study examines several variables and their relationship with neighborhood deprivation to assess predictors of LCS completion rates. As previously stated, to our knowledge, this is the first study to assess the association of the ADI with LCS completion in a primary care context. This study moves beyond the individual-level and evaluates factors that are associated with neighborhood context. Another strength of this study includes utilizing data from a diverse population. The health system in this study cares for a nationally representative patient population of those eligible for LCS [15, 55, 58].

Our study is not without limitations. Due to our use of the EHR, we were limited to the variables collected in the EHR and potential inaccuracies in the data, including missing or misreported variables related to smoking history. For example, approximately 62% of the LCS orders either had a site listed not affiliated with the health system (i.e., community radiology location, Advanced Radiology) or the performing location was missing on the order form in the EHR. Due to this missing data, we used the 90 locations (seven locations were affiliated with the health system) from the ACR LCS locator tool in the analysis. Furthermore, due to the missing EHR data (Table 1), we were unable to assess the total sample for LCS eligibility. We were also unable to utilize the EHR to determine if patients were aware if an LCS order had been placed for them, although based on the typical workflow, patients are given a printout of the LCS order form during the clinical encounter. Additionally, we were unable to address the influence of comorbidities in our analysis due to a high percentage of incomplete data. Although published literature demonstrates that patient characteristics and neighborhood disadvantage have complex interrelationships that impact cancer screening behavior and outcomes [59–61], our study had insufficient sample size to examine interactions between these variables.

Our data is from solely one health system with patients primarily living in Maryland and D.C. which limits generalizability to other primary care practices in other regions of the United States. However, this integrated health system is the largest healthcare provider in Maryland and D.C., so we anticipate the results are likely representative of our catchment area. Finally, while the ADI is a commonly used measure of neighborhood SES, alternative measures of neighborhood SES or even multiple measures could better characterize the area-level factors that influence LCS. Future studies could examine interventions such as patient navigation to help patients schedule their LCS to the nearest site, transportation support, and mobile LCS programs to increase the LCS uptake among patients who live in socioeconomically disadvantaged neighborhoods.

## Conclusions

Data from a large, socioeconomically diverse health system demonstrated how higher neighborhood disadvantage to nearest LCS site was associated with lower LCS completion among patients referred to screening. Primary care practices, with their focus on cancer screenings and key role as often being the first point of contact for LCS in the health care system, may be an effective setting to develop and implement quality improvement initiatives that consider neighborhood context to increase successful completion of LCS.

## Supplementary Information

Supplementary Material 1.

## Data Availability

The dataset and analysis for the patient-level demographic, clinical, and address variables during the current study are not publicly available due to the inclusion of protected health information under HIPAA.The ADI dataset downloaded for use in the current study are available from the University of Wisconsin at https://www.neighborhoodatlas.medicine.wisc.edu/. Kind AJH, Buckingham WR. Making Neighborhood-Disadvantage Metrics Accessible — The Neighborhood Atlas. N Engl J Med. 2018;378(26):2456-2458. doi: 10.1056/NEJMp1802313.

## Abbreviations

U.S.: United States
LCS: Lung cancer screening
NELSON: Nederlands–Leuvens Longkanker Screenings Onderzoek
SES: Socioeconomic status
ADI: Area Deprivation Index
EHR: Electronic Health Record
D.C.: District of Columbia
GEE: Generalized Estimating Equations
ACR: American College of Radiology
OR: Odds ratio
CI: Confidence interval
SD: Standard deviation
