# Supplementary material for: Lung cancer screening completion rates and neighborhood disadvantage among primary care patients in an integrated delivery network health system: a retrospective data analysis
Source: BMC Prim Care. 2026 May 27;27:283. doi: 10.1186/s12875-026-03395-2 (PMC13418439; doi:10.1186/s12875-026-03395-2)
Supplement: Supplementary file 1 — Supplementary Material 1. [file 12875_2026_3395_MOESM1_ESM.docx]

|  | **ADI** | | | | | |  |
| --- | --- | --- | --- | --- | --- | --- | --- |
|  | **Low (N=262)** | | **Medium (N=228)** | | **High (N=276)** | |  |
| **Characteristics** | **N** | **%** | **N** | **%** | **N** | **%** | **Total (N=766)** |
| **Age** | | | | | | | |
| Years, M (SD) | 66 (7) | | 65 (7) | | 65 (6) | | 65 (7) |
| **Sex** | | | | | | | |
| Female | 119 | 31% | 119 | 31% | 150 | 38% | 388 |
| Male | 143 | 38% | 109 | 29% | 126 | 33% | 378 |
| **Race** | | | | | | | |
| Black | 60 | 22% | 83 | 31% | 125 | 47% | 268 |
| White | 173 | 39% | 132 | 30% | 142 | 32% | 447 |
| Other | 29 | 57% | 13 | 25% | 9 | 18% | 51 |
| **Insurance** | | | | | | | |
| Government/Medicaid/Medicare/MCO | 130 | 44% | 132 | 45% | 171 | 58% | 293 |
| Commercial/Private | 124 | 29% | 124 | 29% | 81 | 19% | 433 |
| Self pay/other | 2 | 13% | 1 | 7% | 12 | 80% | 15 |
| Missing | 6 |  | 7 |  | 12 |  | 25 |
| **Clinic Type** | | | | | | | |
| Hospital-based/Resident Clinic | 53 | 22% | 80 | 33% | 113 | 46% | 246 |
| Community-based | 209 | 40% | 148 | 28% | 163 | 31% | 520 |
| **State of Residence** | | | | | | | |
| Maryland (MD) | 215 | 34% | 191 | 31% | 220 | 35% | 626 |
| District of Columbia (DC) | 47 | 34% | 37 | 26% | 46 | 33% | 140 |
| **Area-level Variables** | | | | | | | |
| Euclidean Distance (Miles), M (SD) | 6 (4) | | 6 (4) | | 5 (3) | | 6 (4) |
| Road Network Distance (Miles), M (SD) | 5 (4) | | 8 (5) | | 6 (3) | | 7 (4) |
| Travel Minutes to Nearest LCS Site, M (SD) | 18 (9) | | 21 (10) | | 21 (9) | | 20 (9) |

**Supplemental Material 1.** Distribution of participant characteristics by ADI Categories
